# Combined Straw and Plastic Film Mulching Can Increase the Yield and Quality of Open Field Loose-Curd Cauliflower

**DOI:** 10.3389/fnut.2022.888728

**Published:** 2022-04-29

**Authors:** Yandong Xie, Jinwu Li, Li Jin, Shouhui Wei, Shuya Wang, Ning Jin, Junwen Wang, Jianming Xie, Zhi Feng, Guobin Zhang, Jian Lyu, Jihua Yu

**Affiliations:** ^1^College of Horticulture, Gansu Agricultural University, Lanzhou, China; ^2^Gansu Provincial Key Laboratory of Arid Land Crop Science, Gansu Agricultural University, Lanzhou, China

**Keywords:** loose-curd cauliflower, straw mulch, yield, nutritional quality, volatile compounds, HS-SPME-GC-MS

## Abstract

To evaluate the impact of straw mulching on the production of open field loose-curd cauliflower, this study analyzed the “Feicui No.9” cauliflower variety, grown in field trials in Northwest China, in 2019 and 2020. Plots in an open field were prepared without mulch (CK1) and with plastic film mulch (CK2), as experimental controls, along with three experimental mulching methods, including dual straw and plastic film mulch (T1), inter-row straw mulch (T2), and full straw mulch (T3). The effects of the different ground cover alternatives on loose-curd cauliflower's dry matter accumulation, yield, quality, and volatile compounds, were explored. The results showed that, compared with CK2 treatment, T1 treatment promoted the accumulation of dry matter, and increased the economic and biological yield, by 12.98 and 6.51%, respectively. The soluble sugar and vitamin C content in loose-curd cauliflower heads, subjected to T1 treatment, increased by 18.46 and 8.12%, respectively, and the nitrate content decreased by 25.57%. Moreover, the T1, T2, and T3 treatments significantly increased the levels of macro-, meso-, and microelements. Headspace solid-phase microextraction-gas chromatography-mass spectrometry (HS-SPME-GC-MS) was used to determine the volatile substances in loose-curd cauliflower heads from the 2020 harvesting period. Detected compounds included 17 aldehydes, 15 ketones, 10 alcohols, 15 esters, 29 hydrocarbons, 12 nitrogen-containing compounds, and 17 other substances. T1, T2, and T3 treatments increased the volatile substance content, whereas T1 treatment increased the quantity of volatile substances. In summary, dual mulching with straw and plastic film could promote dry matter accumulation, significantly increase the yield and quality, and effectively improve the flavor of loose-curd cauliflower. This mulching technique can be applied to open field vegetable and corn production areas, providing technical and theoretical support for the realization of high-yield, high-quality production models and a new straw recycling method.

## Introduction

Loose-curd cauliflower (*Brassica oleracea var. botrytis* L.)—also known as loose cauliflower, organic cauliflower, and pine cauliflower—has a delicious flavor and is rich in soluble sugar, vitamin C, crude protein, mineral elements, and other nutrients. It is reportedly able to resist cancer, plays a role in cancer prevention, and is well-loved by consumers (1–3). Loose-curd cauliflower is one of the main varieties of plateau summer vegetables, of which the Lanzhou area is a main producer. Lanzhou experiences sufficient sunshine in summer and a large temperature difference between day and night, representing natural advantages for producing plateau summer vegetables. The plateau summer vegetable industry has become a main source of income for farmers in Lanzhou City (4), Gansu Province, is located in an arid/semi-arid area of the Loess Plateau. The ecological environment is severe, characterized by soil erosion and deficient water resources. In addition, long-term excessive application of chemical fertilizers and mulch film pollution has caused a series of problems such as soil and vegetable quality decline, and yield reduction, severely restricting the development of the vegetable industry (5, 6). Therefore, improving the soil cultivation environment is of great significance for the restoration of soil productivity.

Northwest China is rich in straw resources, but the straw recycling rate is low, and straw burning is a common phenomenon (7). This practice produces many harmful gases, causes serious air pollution, and severely threatens human health (8–12). The application of straw returning technology can avoid the environmental problems caused by straw burning, while also effectively reducing soil water evaporation, enhancing the rainwater infiltration rate, increasing soil water storage, improving soil structure and microbial diversity and abundance, enhancing soil enzyme activity, improving soil fertility, promoting crop quality, and facilitating improved crop yield and water use efficiency (13–20). Straw contains a large amount of N, P, K, nutrients, and organic matter, which can be used as fertilizer resources during crop growth (21). Therefore, returning straw to the field is conducive to the sustainable development of agriculture. Moreover, plastic film mulching can inhibit the evaporation of water from the soil, preserve moisture, and promote the accumulation of dry matter in crops, thereby increasing the yield and effective use of water (22, 23).

To date, research on straw mulching has mainly focused on food crops such as potato (24), corn (25), and wheat (26), and few reports on research related to vegetable crops, are available. Therefore, in this study, the loose-curd cauliflower variety, “Feicui No. 9,” was used as test material and plots in an open field prepared, both without mulch (CK1) and with plastic film mulch (CK2), as controls, alongside other mulching combinations. The effects of different ground mulching patterns on the yield, quality, and volatile compounds of open field loose-curd cauliflower produced in Lanzhou, were evaluated. The study findings provide a technical and theoretical basis for realizing high-quality production of open field vegetables, and the method of recycling crop straw.

## Materials and Methods

### Plant Materials and Experimental Design

Loose-curd cauliflower (*Brassica oleracea var. botrytis* L.), of the “Feicui No. 9” variety, corn stalk straw, and transparent mulching film were used in this experiment. Fertilizers used included diammonium phosphate (*N* ≥ 18.0%, *P* ≥ 46.0%) (Hubei Sanning Chemical Co., Ltd., Yichang, China), calcium ammonium nitrate (*N* ≥ 15.5%, NO${}_{3}^{-}$ ≥ 14.4%, CaO ≥ 25.5%) (Shanxi Sanxi Chemical Co., Ltd., Taiyuan, China), and Nitro 103 (*N* ≥ 15.0%, *P* ≥ 6.0%, *K* ≥ 21.0%) (Woft Company, City, Country). Corn stalks—amounting to 6,000 kg·ha^−1^–treated in the same way as in 2019, were returned to the field, using rotary tillage.

**Figure 1:**
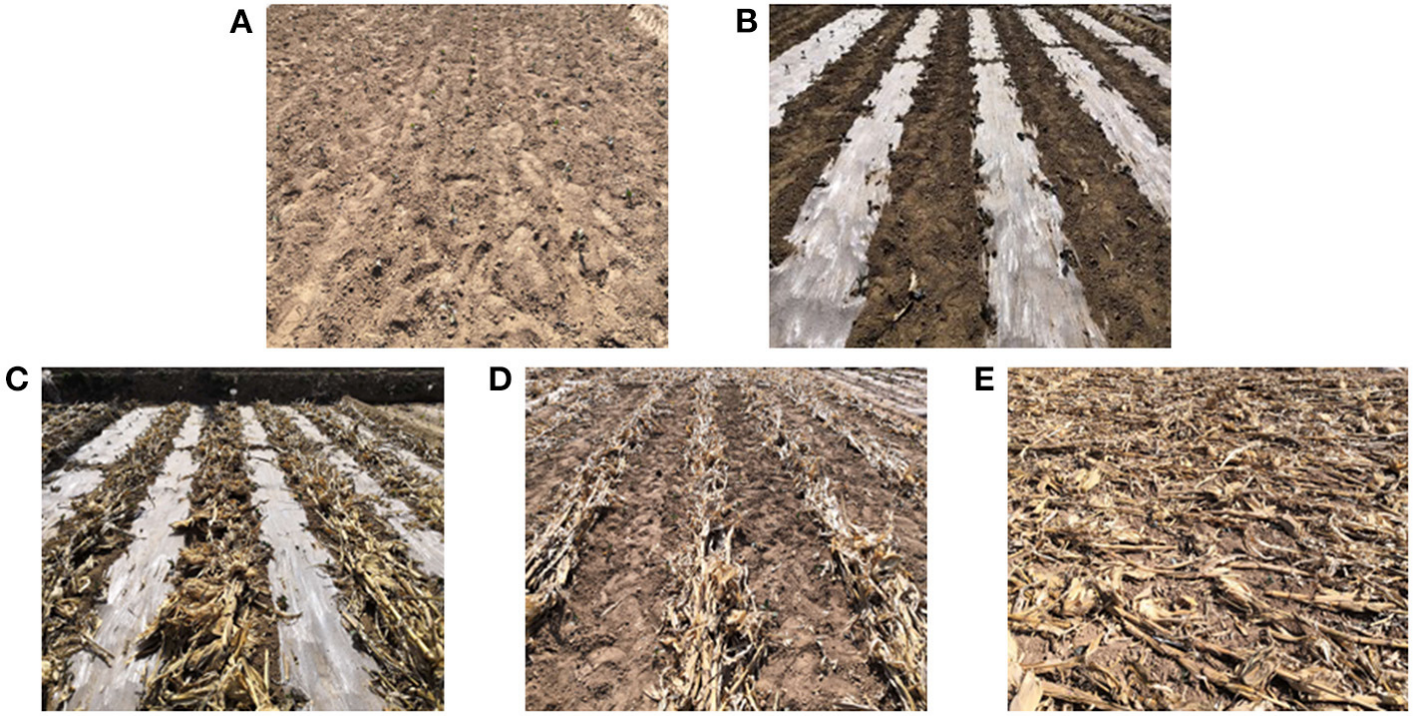
Different mulching patterns applied in this experiment. **(A)** Open field without mulching (CK1): No mulching was applied on ridges or in furrows. **(B)** Plastic film mulch (CK2): transparent plastic film was mulched on the ridges, but no mulching was applied in the furrows. **(C)** Dual mulching with straw and mulching film (T1): ridges were covered with transparent mulching film, whereas corn stalks evenly covered the furrows. **(D)** Straw mulch between rows (T2): no plastic film mulch was applied on the ridges, and furrows were evenly covered with corn stalks. **(E)** Full straw coverage (T3): All ridges and furrows were evenly covered with corn stalks. In all plots, loose-curd cauliflower was planted on the ridges.

The experiment was conducted in Qingshuiyi Township (35°87′N, 104°23′E), Yuzhong County, Lanzhou City, Gansu Province, China, from July to October in 2019 and 2020, respectively. The test area has an average altitude of 1,790 m, annual average temperature of 6°C, a frost-free period of ~100–140 days, average precipitation of 300–400 mm, and multi-year average evaporation of 1343.1 mm. The effective rainfall in this semi-arid area, is 88.8 mm. The test soil was loam, of which the basic physical and chemical properties are shown in Table 1. A total of five treatment plots (Figure 1) were set up in the experiment: open field without mulch (CK1) and with plastic film mulch (CK2), as controls, along with three variations containing straw, which were dual mulch (T1), inter-row straw mulch (T2), and full straw mulch (T3). Each treatment was repeated three times, in a random block arrangement, of which cell length and width were 8.8 and 6.0 m, respectively, equating to a cell area of 52.8 m^2^. In total, 15 plots were prepared, of which each adopted a ridge and double furrow planting arrangement; ridge and furrow width were 70 and 45 cm, respectively, and plants were spaced 60 cm apart. Similar amounts of fertilizer were applied to each treatment plot. The total amounts of fertilizer applied, were as follows: nitrogen fertilizer (*N*), 368.06 kg·ha^−1^, phosphate fertilizer (P_2_O_5_), 495.3 kg·ha^−1^, and potassium fertilizer (K_2_O), 163.8 kg·ha^−1^.

**Table 1:** Main physical and chemical properties of soil in the arable layer of the experimental site.

**Bulk density /(g·cm^**−3**^)**	**Total nitrogen /(g·kg^**−1**^)**	**Total phosphorus /(g·kg^**−1**^)**	**Total potassium /(g·kg^**−1**^)**	**Available phosphorus /(mg·kg^**−1**^)**	**Available potassium /(mg·kg^**−1**^)**	**pH**	**EC (μS·cm^**−1**^)**
1.45	0.16	0.72	22.42	107.34	91.20	7.97	510

### Yield and Dry Matter

During the loose-curd cauliflower's rosette, heading, and harvesting stages, five plants of uniform size were selected from each plot, and the dry matter accumulation measured. The samples were placed in an oven at 105°C for 30 min, and then dried to a constant weight at 80°C, before each part was weighed to determine the dry weight. During the harvesting period, 10 loose-curd cauliflower heads were randomly selected from the center of each plot (to remove the marginal effect), and the single head weight determined using an electronic scale, from which the economic and biological yield were calculated, and finally converted into a hectare yield (27).

### Quality Indicators

To determine nutritional quality, three cauliflower heads of uniform size were selected from each plot during the harvesting period. Then, one quarter of each head was randomly selected, chopped, and mixed together, for quality determination. Soluble sugar content was determined using the anthrone colorimetric method (28) and soluble protein content, using the Coomassie brilliant blue method (29), whereas vitamin C and nitrate content were determined using the 2,6-dichloroindophenol stain (30) and salicylic acid methods (31), respectively.

To determine the mineral element content, cauliflower heads were selected during harvest, dried in an oven, ground in a pulverizer, passed through a 2 mm sieve, and placed in a Ziploc bag. Molybdenum blue colorimetry and a UV 1780 spectrophotometer (Shimadzu Instruments Co., Ltd., Suzhou, China) were used to determine the phosphorus content (32). Further, the potassium content was measured using the flame spectrophotometer method and an AP1302 flame photometer (Shanghai Aopu Analytical Instrument Company, Shanghai, China) (33). Finally, levels of calcium, magnesium, copper, manganese, iron, and zinc were measured using a ZEEnit-700P atomic absorption spectrophotometer (Analytik Jena GmbH, Jena, Germany) (34).

### Instruments and Equipment

Instruments used in this study included: a DF-101S heat-collecting magnetic stirrer (Zhengzhou Yarong Instrument Co., Ltd., Zhengzhou, China), a solid-phase microextraction (SPME) syringe, a 75 μm carboxen-polydimethylsiloxane (CAR/PDMS) SPME extraction head (Supelco, Inc., Bellefonte, PA, USA), a DB-WAX elastic quartz capillary column (20 m, 0.18 mm, 0.18 μm, Agilent, Santa Clara, CA, USA), and a gas chromatography-mass spectrometer for GC-MS (Thermo Fisher Scientific, Waltham, MA, USA).

### Volatile Compounds

#### Volatile Compounds Extraction

The volatile compounds in loose-curd cauliflower heads, obtained during the 2020 harvesting period. Furthermore, on the basis of previous studies, this study further the volatile compounds extraction procedure of loose-curd cauliflower heads in the Gansu Provincial Key Laboratory of Arid Land Crop Science, Gansu Agricultural University focusing on improving the accuracy of volatiles measurements (35). First, the sample was ground, where after 5 g of the homogenized sample was accurately and quickly weighed into a 15 mL headspace bottle, to which 1.25 g of anhydrous Na_2_SO_4_ and 30 μL of 82.1 mg/L 2-octanol internal standard sample, were added. The mixture was magnetically stirred and the cap immediately tightened, before the bottle was placed in a 60°C thermostatic magnetic stirrer. The solution was equilibrated at a rate of 500 r/min for 10 min, extracted and absorbed at 60°C for 30 min, and immediately inserted into the gasification chamber, where it was analyzed for 5 min.

Gas chromatography (GC) conditions were as follows: DB-1701 elastic quartz capillary column (30 m, 0.25 mm, 0.25 μm); inlet temperature: 250°C; carrier gas: high-purity helium (purity ≥ 99.999%); flow rate: 1.0 mL/min; sampling method: splitless injection; programmed temperature rise: initial temperature of 40°C, rising to 190°C at 3.5°C/min, maintained for 3 min.

Mass spectrometry (MS) conditions: electron ionization (EI); electron energy: 70 eV; ion source temperature: 200°C; transmission line temperature: 190°C; scan mode: full scan; scan quality range: 35–500 u.

#### Volatile Compounds Analysis

The Automated Mass Spectral Deconvolution and Identification System (AMDIS) and the mass spectrum library (NIST2014) were used to search and analyze data, and only substances with a mass spectrum matching score >70%, were retained. The formula for calculating the volatile matter content in loose-curd cauliflower heads, was as follows:


(1) $$\begin{array}{r} \text{C}=\left(\text{S}1/\text{S}2\right)\;\times \left(\text{M}1/\text{M}2\right)\;\times 1000 \end{array}$$

where C represents the relative concentration of volatile compounds (μg·kg^−1^), S1 and S2 represent the peak area measured by the sample and the peak area of the internal standard, respectively, and M1 and M2 represent the quality of the internal standard and the sample, respectively.

### Statistical Analysis

Microsoft Excel 2019 (Microsoft Corp., Redmond, WA, USA) was used to sort and graphically represent the experimental data. SPSS software, version 20.0 (IBM Corp., Armonk, NY, USA) was used for variance analysis (Duncan, *p* < 0.05) and conducting Duncan's new Multiple Range Test (*p* < 0.05). Principle Component Analysis (PCA) score scatter and PC loading plots were constructed using SPSS version 23.0 (IBM Corp.) and OriginPro 8.5.0 (OriginLab Corporation, Northampton, MA, USA).

## Results

### Effects of Different Ground Mulching Patterns on the Dry Weight of Loose-Curd Cauliflower

As shown in Table 2, dry matter accumulation—represented by shoot and root dry weights, respectively—was highest in cauliflower subjected to T1 treatment, followed by those grown under CK2 treatment, whereas T3 treatment resulted in the lowest values, in 2019. However, in 2020, T1 treatment significantly promoted the accumulation of dry matter, during the rosette, heading, and harvesting stages. Compared with cauliflower grown under CK2 treatment, the average shoot and root dry weights of those under T1 treatment increased by 42.74 and 23.22%, respectively, during the rosette stage, and 9.58 and 23.92%, respectively, during the heading stage. Average shoot dry weight of cauliflower grown under T1 treatment an increasing trend at first during harvesting time, compared with those under CK2 treatment, but there was no significant difference in root dry weight between the treatments.

**Table 2:** Effects of different ground mulching patterns on the dry weight of loose-curd cauliflower.

		**Rosette stage**		**Heading stage**		**Harvesting stage**	
**Year**	**Treatment**	**Shoot dry weight/(g)**	**Root dry weight/(g)**	**Shoot dry weight/(g)**	**Root dry weight/(g)**	**Shoot dry weight/(g)**	**Root dry weight/(g)**
2019	CK1	10.15 ± 0.55b	0.64 ± 0.01c	127.42 ± 4.40b	8.70 ± 0.04c	198.89 ± 8.77b	15.84 ± 1.19bc
	CK2	13.98 ± 0.26a	0.85 ± 0.08ab	169.44 ± 7.21a	10.96 ± 0.27ab	264.27 ± 4.60a	16.72 ± 0.53b
	T1	13.88 ± 1.48a	0.96 ± 0.03a	173.43 ± 4.40a	12.15 ± 0.91a	271.59 ± 12.11a	20.50 ± 0.32a
	T2	11.81 ± 0.86b	0.81 ± 0.04b	138.32 ± 6.59b	10.39 ± 0.14b	246.64 ± 6.13a	16.10 ± 0.80bc
	T3	8.89 ± 0.44b	0.59 ± 0.02c	124.76 ± 3.89b	10.04 ± 0.52bc	209.91 ± 4.96b	13.98 ± 0.58c
2020	CK1	78.13 ± 1.33b	4.22 ± 0.15c	227.58 ± 4.75bc	10.85 ± 0.91b	241.83 ± 9.57b	11.82 ± 0.59a
	CK2	104.34 ± 6.15ab	5.88 ± 0.26b	242.91 ± 2.54b	10.97 ± 0.28b	228.74 ± 5.31b	12.44 ± 0.22a
	T1	148.93 ± 36.76a	7.25 ± 0.27a	266.19 ± 3.31a	13.59 ± 1.01a	276.55 ± 6.32a	13.75 ± 0.11a
	T2	75.26 ± 7.74b	5.14 ± 0.38b	214.90 ± 13.54c	11.13 ± 0.24b	240.25 ± 1.80b	12.69 ± 0.68a
	T3	71.03 ± 4.13b	4.01 ± 0.17c	214.21 ± 4.80c	10.70 ± 0.48b	226.53 ± 15.00b	11.54 ± 1.23a

*Values are expressed as mean ± SE (n = 3). Different letters denote significant differences (p < 0.05). CK1, CK2, T1, T2, and T3 are defined in Figure 1*.

### Effects of Different Ground Mulching Patterns on Loose-Curd Cauliflower Yield

Table 3 reveals that the head weight, economic and biological yield, and economic coefficient of loose-curd cauliflower grown in soil subjected to the different treatments, showed a trend of increasing at first and then decreasing. Compared with those under CK2 treatment, the average economic and biological yield of cauliflower grown in soil subjected to T1 treatment increased by 12.98 and 6.51%, respectively. The head weight was highest in cauliflower from T1-treated soil, whereas the head weight, and both economic and biological yield of cauliflower from soil subjected to T2 and T3 treatments, were lower than those grown under CK1 and CK2 treatments. There was no significant difference in the economic coefficient of cauliflower grown in either T1- or CK2-treated soil.

**Table 3:** Effects of different ground mulching patterns on loose-curd cauliflower yield.

**Year**	**Treatment**	**Head weight**	**Economic yield**	**Biomass yield**	**Economic coefficient/(%)**
		**(kg)**	**(kg·ha^**−1**^)**	**(kg·ha^**−1**^)**	
2019	CK1	1.21 ± 0.82b	33,726.73 ± 2,289.17b	80,744.10 ± 788.90a	0.42 ± 0.03b
	CK2	1.40 ± 0.07ab	39,692.68 ± 1,328.42ab	82,603.01 ± 2,940.60a	0.48 ± 0.01ab
	T1	1.54 ± 0.03a	43,027.68 ± 885.74a	84,573.13 ± 1,986.31a	0.51 ± 0.01a
	T2	1.35 ± 0.12ab	36,932.04 ± 2,907.73ab	78,255.16 ± 4,298.02ab	0.47 ± 0.02ab
	T3	1.19 ± 0.06b	33,121.49 ± 1,652.69b	71,010.80 ± 1,948.57b	0.47 ± 0.01ab
2020	CK1	1.20 ± 0.05c	33,226.48 ± 14,89.52c	94,800.46 ± 2,194.41c	0.35 ± 0.02b
	CK2	1.42 ± 0.02b	39,525.93 ± 551.53b	103,137.96 ± 724.19b	0.38 ± 0.06ab
	T1	1.68 ± 0.05a	46,720.88 ± 1,521.82a	114,100.23 ± 1,526.51a	0.41 ± 0.02a
	T2	0.84 ± 0.16d	23,345.00 ± 1,536.94d	94,028.47 ± 2,609.97c	0.25 ± 0.02c
	T3	0.93 ± 0.05d	25,784.49 ± 1,430.50d	94,399.03 ± 1,917.65c	0.27 ± 0.01c

*Values are expressed as the mean ± SE (n = 3). Different letters denote significant differences (p < 0.05). CK1, CK2, T1, T2, and T3 are defined in Figure 1*.

### Effects of Different Ground Mulching Patterns on Nutritional Quality of Loose-Curd Cauliflower Heads

**Figure 2:**
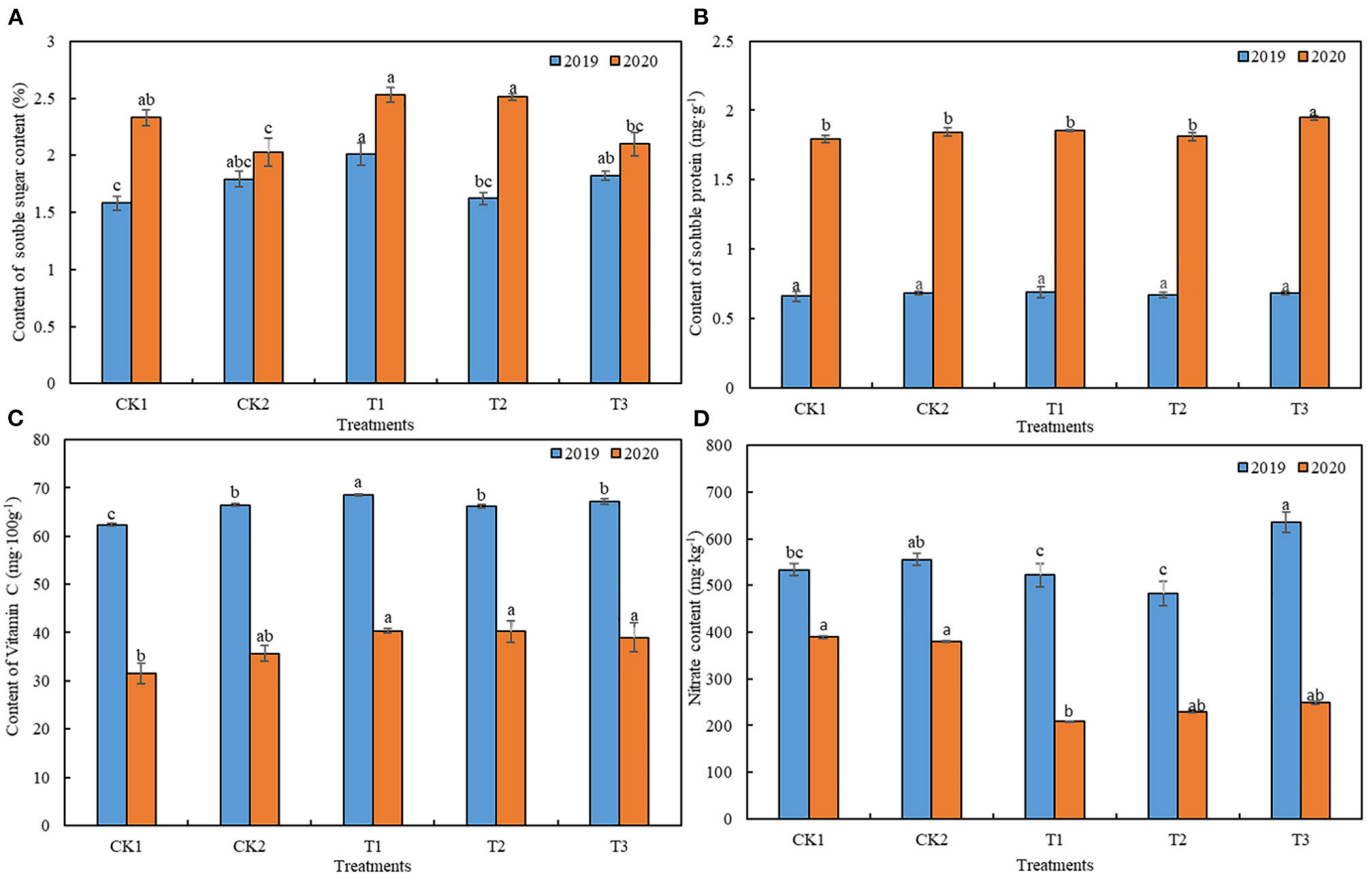
Effects of different ground mulching patterns on **(A)** soluble sugar, **(B)** soluble protein, **(C)** vitamin C, and **(D)** nitrate contents in loose-curd cauliflower heads. Values are expressed as mean ± SE (*n* = 3). Different letters denote significant differences (*p* < 0.05). CK1, CK2, T1, T2, and T3 are defined in Figure 1.

T1 treatment significantly improved the nutritional quality of loose-curd cauliflower, as shown in Figure 2. Compared with cauliflower grown under CK1 and CK2 treatments, the average soluble sugar content in those grown under T1 treatment, increased by 17.90 and 18.62%, respectively (Figure 2A), while no difference was observed in soluble protein content (Figure 2B). Moreover, average vitamin C content increased by 18.92 and 8.12%, respectively (Figure 2C), and average nitrate content decreased by 24.28 and 25.57%, respectively (Figure 2D).

### Effects of Different Ground Mulching Patterns on Mineral Elements in Loose-Curd Cauliflower Heads

As shown in Table 4, the straw mulching treatment significantly affected the mineral element content in loose-curd cauliflower. Compared with the CK2 treatment, T1, T2, and T3 treatments significantly induced accumulation of macro- (P, K), meso- (Ca, Mg), and micro- (trace) elements (Fe, Mn, and Zn) in loose-curd cauliflower. Mineral element levels increased the most in cauliflower grown in T3-treated soil; on average, P, K, Ca, Mg, Fe, Mn, and Zn increased by 32.01, 9.22, 27.08, 14.12, 26.33, 10.76, and 40.23%, respectively. Conversely, mineral element content was lowest in cauliflower grown in CK1-treated soil. These findings indicated that straw mulching could promote the absorption of mineral elements in loose-curd cauliflower heads, to a certain extent.

**Table 4:** Effects of different ground mulching patterns on the mineral element content in loose-curd cauliflower heads.

**Year**	**Treatment**	**P**	**K**	**Ca**	**Mg**	**Fe**	**Mn**	**Zn**
		**g·kg^**−1**^**	**g·kg^**−1**^**	**g·kg^**−1**^**	**g·kg^**−1**^**	**mg·kg^**−1**^**	**mg·kg^**−1**^**	**mg·kg^**−1**^**
2019	CK1	3.24 ± 0.09d	32.02 ± 1.22c	1.00 ± 20.34d	5.35 ± 0.004c	234.90 ± 4.77c	30.74 ± 0.68c	28.24 ± 0.66c
	CK2	3.70 ± 0.03cd	33.57 ± 1.35bc	1.30 ± 35.34c	5.36 ± 0.009c	241.17 ± 11.18bc	31.40 ± 0.71bc	29.32 ± 0.94c
	T1	3.91 ± 0.08bc	35.33 ± 1.35abc	1.60 ± 66.24b	5.43 ± 0.006b	259.70 ± 4.92ab	33.01 ± 0.56ab	30.08 ± 0.61bc
	T2	4.46 ± 0.30ab	36.61 ± 0.56ab	1.37 ± 75.40c	5.48 ± 0.009a	266.87 ± 5.13a	33.91 ± 0.75a	32.03 ± 0.93ab
	T3	4.60 ± 0.25a	38.58 ± 0.95a	1.80 ± 35.17a	5.49 ± 0.008a	281.43 ± 5.58a	35.02 ± 0.20a	32.77 ± 0.32a
2020	CK1	5.25 ± 0.48bc	30.75 ± 0.22ab	1.07 ± 0.09b	2.44 ± 0.040a	158.40 ± 3.13c	79.91 ± 1.26c	70.11 ± 1.25c
	CK2	4.61 ± 0.36c	30.35 ± 0.05b	1.21 ± 0.01ab	2.13 ± 0.110b	180.60 ± 32.46bc	79.64 ± 1.71c	73.23 ± 9.02c
	T1	6.27 ± 0.13ab	31.33 ± 0.14a	1.42 ± 0.05a	2.58 ± 0.050a	256.37 ± 12.30a	83.58 ± 0.82b	100.21 ± 2.22b
	T2	5.65 ± 0.06abc	31.22 ± 0.48a	1.24 ± 0.01ab	2.60 ± 0.090a	227.40 ± 7.26ab	87.93 ± 0.84a	80.48 ± 0.84c
	T3	6.44 ± 0.37a	31.42 ± 0.16a	1.40 ± 0.11a	2.68 ± 0.040a	245.57 ± 3.18a	87.60 ± 0.66a	123.53 ± 5.91a

*Values are expressed as the mean ± SE (n = 3). Different letters denote significant differences (p < 0.05). CK1, CK2, T1, T2, and T3 are defined in Figure 1. p, Phosphorus; K, Potassium; Ca, Calcium; Mg, Magnesium; Fe, Iron; Mn, Manganese; Zn, Zinc*.

### Effects of Different Ground Mulching Patterns on the Flavor Quality of Loose-Curd Cauliflower Heads

As shown in Table 5, 115 volatile compounds were detected in loose-curd cauliflower heads, using the HS-SPME- GC-MS methodology. These included 17 aldehydes, 15 ketones, 10 alcohols, 15 esters, 29 hydrocarbons, 12 nitrogen-containing compounds—mainly comprising nitriles—and 17 other compound species, which were mainly ethers, phenols, and furans, among others. The number of volatile compounds detected was highest in cauliflower grown in T1-treated soil, reaching 50 species, followed by those grown in soil subjected to CK2 treatment, with 48 varieties. Cauliflower grown in T2- and T3-treated soil contained the least volatile compounds, at 45 varieties each. Overall, T1 soil treatment resulted in the highest total volatile substance content—which was 6867.59 μg/kg—with T3 treatment in second place, at 5169.63 μg/kg, and CK1 treatment ranking last, at 4209.15 μg/kg. Compared with those grown in soil subjected to CK1 and CK2 treatments, the total volatile substance content in loose-curd cauliflower heads from T1-, T2-, and T3-treated soil, showed significant increases of 63.24 and 54.19%, 13.9 and 7.6%, and 22.82 and 16.07%, respectively. The most abundant volatile compound was (E)-2-hexenal, of which the content was 1565.9 μg/kg.

**Table 5:** Effects of different ground mulching patterns on volatile compounds in loose-curd cauliflower heads.

**Group (No.)**	**Volatile compounds**	**Chemical formula**	**Retention time/min**	**Content (μg/kg)**					**CAS**
				**CK1**	**CK2**	**T1**	**T2**	**T3**	
**Aldehydes**									
1	1,5-Pentanedial	C_5_H_8_O_2_	7.04	—	—	250.22	—	—	111-30-8
2	1-Hexanal	C_6_H_12_O	7.10	—	—	157.69 ± 8.15a	20.02 ± 1.51b	—	66-25-1
3	(E)-2-Hexenal	C_6_H_10_O	9.96	—	1,134.50 ± 11.93c	1,404.27 ± 61.71b	649.32 ± 61.15d	1,565.90 ± 131.23a	6728-26-3
4	4-Methyl-3-pentenal	C_6_H_10_O	9.98	—	—	—	1112.77	—	5362-50-5
5	2,6-Dimethyl-5-heptenal	C_9_H_16_O	10.41	116.65 ± 7.60b	—	—	—	524.09 ± 30.15a	106-72-9
6	2-Propenal	C_3_H_4_O	10.74	—	—	31.57 ± 5.96b	38.16 ± 5.57a	—	107-02-8
7	Benzaldehyde	C_7_H_6_O	14.02	—	—	36.47 ± 3.07c	50.85 ± 2.17b	60.77 ± 3.10a	100-52-7
8	(E)-4-Oxohex-2-enal	C_6_H_8_O_2_	15.88	—	—	32.88	—	—	1000374-04-2
9	(E,E)-2,4-Heptadienal	C_7_H_10_O	16.00	19.76 ± 1.45d	35.42 ± 1.75c	47.71 ± 6.59bc	59.38 ± 3.40b	83.57 ± 5.46a	4313-03-5
10	2-Methyl-3-methylene-cyclopentanecarboxaldehyde	C_8_H_12_O	16.58	—	—	—	—	8.32	97663-70-2
11	(E)-2-Octenal	C_8_H_14_O	16.96	16.78 ± 1.19c	16.13 ± 1.28c	36.62 ± 5.22a	25.30 ± 1.39b	30.64 ± 1.65ab	2548-87-0
12	Benzeneacetaldehyde	C_8_H_8_O	17.18	10.56 ± 0.51b	—	23.46 ± 1.59a	—	19.24 ± 1.51a	122-78-1
13	1-Nonanal	C_9_H_18_O	17.59	96.40 ± 4.80c	74.15 ± 4.45c	169.21 ± 7.83b	209.45 ± 11.46a	176.20 ± 6.12b	124-19-6
14	3-Ethyl-benzaldehyde	C_9_H_10_O	19.96	20.70 ± 1.59bc	16.37 ± 1.12c	31.25 ± 2.14a	24.43 ± 2.15b	35.06 ± 3.03a	34246-54-3
15	1-Decanal	C_10_H_20_O	20.13	16.46 ± 1.57c	10.68 ± 0.58d	32.30 ± 2.12a	18.47 ± 1.54bc	22.45 ± 1.57b	112-31-2
16	(E,E)-2,4-Decadienal	C_10_H_16_O	24.72	—	6.93 ± 0.52c	18.78 ± 0.89a	12.67 ± 1.34b	—	25152-84-5
17	(Z)-4,5-Epoxy-2-decenal	C_10_H_16_O_2_	24.82	—	—	32.47	—	—	1000360-26-2
**Ketones**									
18	3-Hydroxy-2-butanone	C_4_H_8_O_2_	1.59	368.42 ± 22.65a	—	—	—	114.79 ± 2.57b	513-86-0
19	3-Pentanone	C_5_H_10_O	7.56	—	19.81	—	—	—	96-22-0
20	Ethanone, 2-hydroxy-1,2-diphenyl	C_14_H_12_O_2_	8.06	—	—	—	14.38	—	119-53-9
21	3-Methyl-cyclopentanone	C_6_H_10_O	9.97	454.38 ± 16.39b	662.36 ± 20.03a	—	—	—	1757-42-2
22	7-Azabicyclo[4,2,0]octan-8-one	C_7_H_11_NO	10.40	238.85	—	—	—	—	34102-49-3
23	2-Methyl-5-hydroxy-7-methoxy-3-Phenyl-4-chromenone	C_17_H_14_O_4_	11.33	440.91	—	—	—	—	55927-39-4
24	2-(Formyloxy)-1-phenyl-ethanone	C_9_H_8_O_3_	14.02	—	18.20 ± 1.57a	—	—	15.66 ± 1.67b	55153-12-3
25	1-Penten-3-one	C_5_H_8_O	10.72	8.62	—	—	—	—	1629-58-9
26	5-Methyl-4-hexen-3-one	C_7_H_12_O	15.87	—	—	—	8.98	—	13905-10-7
27	1-Phenyl-ethanone	C_8_H_8_O	17.69	—	—	9.92 ± 0.71a	8.49 ± 1.57b	—	98-86-2
28	5-Methyl-1-phenyl-1-hexanone	C_13_H_18_O	17.69	11.73 ± 1.01b	7.35 ± 0.50c	19.09 ± 1.51a	—	—	25552-17-4
29	2-(1,1-Dimethylethyl)-Cyclobutanoe	C_8_H_14_O	17.80	19.56	—	—	—	—	4423-94-3
30	1-Hepten-3-one	C_7_H_12_O	18.67	—	—	16.41	—	—	2918-13-0
31	3-Methyl-2-pentyl-2-cyclopenten-1-one	C_11_H_18_O	21.84	—	—	—	—	13.22	1128-08-1
32	[1,1'-Bicyclohexyl]-2-one	C_12_H_20_O	22.58	—	8.38 ± 1.51b	—	19.35 ± 1.01a	11.72 ± 0.61b	90-42-6
**Alcohols**									
33	(S)-(+)-1,2-Propanediol	C_3_H_8_O_2_	1.53	—	—	365.49	—	—	4254-15-3
34	(E)-4-Hexen-1-ol	C_6_H_12_O	10.39	—	—	472.91	—	—	928-92-7
35	(E)-3-Hexen-1-ol	C_6_H_12_O	10.41	—	—	138.59	—	—	928-97-2
36	1-Hexanol	C_6_H_14_O	10.76	13.07 ± 0.50d	34.53 ± 1.01c	61.76 ± 2.05b	—	95.21 ± 4.10a	111-27-3
37	2,3-Butanediol	C_4_H_10_O_2_	14.75	—	—	276.00	—	—	19132-06-0
38	3-Methyl-3-heptanol	C_8_H_18_O	15.09	—	6.42	—	—	—	5582-82-1
39	2-Methylene cyclopentanol	C_6_H_10_O	16.95	18.61	—	—	—	—	20461-31-8
40	(S)-(+)-5-Methyl-1-heptanol	C_8_H_18_O	18.81	—	5.23 ± 0.60b	22.18 ± 0.99a	—	—	57803-73-3
41	1-Nonanol	C_9_H_20_O	18.91	27.26 ± 1.66b	30.43 ± 0.52b	41.64 ± 2.40a	10.48 ± 0.88c	—	143-08-8
42	(S)-(+)-6-Methyl-1-octanol	C_9_H_20_O	18.91	20.16 ± 0.54b	21.31 ± 1.52a	—	—	—	110453-78-6
**Esters**									
43	Ethyl acetate	C_4_H_8_O_2_	7.06	31.03	—	—	—	—	141-78-6
44	Methyl isothiocyanate	C_2_H_3_NS	7.13	15.31	—	—	—	—	556-61-6
45	2-Phenylethyl docosanoate	C_30_H_52_O_2_	9.66	—	—	—	—	9.97	1000395-18-6
46	(Z)-Hex-3-en-1-yl propyl carbonate	C_10_H_18_O_3_	10.39	226.35	—	—	—	—	1000372-80-5
47	(E,Z)-2-Butenoic acid, 3-hexenyl ester	C_10_H_16_O_2_	10.41	—	—	178.39	—	—	65405-80-3
48	Ethyl (E)-hex-3-enyl carbonate	C_9_H_16_O_3_	10.42	—	—	—	—	440.46	1000373-83-8
49	2-Methylbutyl isothiocyanate	C_6_H_11_NS	16.67	—	7.10 ± 0.52b	11.25 ± 0.58a	—	—	4404-51-7
50	Octyl formate	C_9_H_18_O_2_	17.26	18.65 ± 0.85d	24.61 ± 2.00cd	32.07 ± 2.06c	72.24 ± 3.95a	52.47 ± 6.02b	112-32-3
51	1,3-Benzenediol, monobenzoate	C_13_H_10_O_3_	17.69	7.54 ± 0.34b	14.43 ± 2.62a	—	—	—	136-36-7
52	2-Butenoic acid, 2-methyl-2-methylpropyl ester	C_9_H_16_O_2_	18.69	—	—	—	21.94 ± 2.51a	15.35 ± 0.52b	66917-61-1
53	Butyl acrylate	C_7_H_12_O_2_	20.13	—	14.41 ± 1.06a	—	—	8.77 ± 0.68b	141–32-2
54	3-Methylthiopropyl isothiocyanate	C_5_H_9_NS_2_	23.69	—	—	—	180.94 ± 7.19a	152.75 ± 6.14b	505-79-3
55	Erucin	C_6_H_11_NS_2_	25.70	—	—	—	51.88 ± 2.56b	70.26 ± 6.21a	4430-36-8
56	(2-Isothiocyanatoethyl)-benzene	C_9_H_9_NS	26.06	—	—	—	17.71 ± 1.48b	22.64 ± 2.14a	2257/9/2
57	Ethyl Palmitate	C_18_H_36_O_2_	30.86	12.25 ± 0.64b	12.89 ± 0.65b	23.39 ± 3.04a	—	15.72 ± 0.74b	628-97-7
**Hydrocarbons**									
58	1,3-Dimethyl-benzene	C_8_H_10_	8.32	—	—	—	732.48	—	108-38-3
59	3-Ethyl-1,5-octadiene	C_10_H_18_	11.87	—	—	—	—	44.17	1000114-87-7
60	Methyl ethyl cyclopentene	C_8_H_14_	15.53	—	18.71 ± 2.67c	—	33.99 ± 5.40b	43.62 ± 3.06a	19780-56-4
61	3-Methyl-octane	C_9_H_20_	15.88	—	16.49	—	—	—	2216-33-3
62	5-Methyl-3-heptyne	C_8_H_14_	15.99	—	36.20 ± 1.58b	—	—	89.46 ± 4.28a	61228-09-9
63	1-Ethyl-3-methyl-cyclopentane	C_8_H_16_	16.96	—	—	—	—	20.11	3728-55-0
64	Spiro[2,4]hepta-4,6-diene	C_7_H_8_	17.18	8.63	—	—	—	—	765-46-8
65	Ethyl benzene	C_8_H_10_	17.41	—	—	—	178.75	—	100-41-4
66	Methyl-cyclohexane	C_7_H_14_	17.80	14.24 ± 0.99b	—	17.08 ± 0.57a	—	—	108-87-2
67	(E)-4-Ethyl-2-octene	C_10_H_20_	17.80	—	—	24.76	—	—	74630-09-4
68	(Z)-4-Methyl-2-decene	C_11_H_22_	17.80	30.63 ± 2.45a	26.06 ± 2.62b	—	—	—	74630-30-1
69	Dodecane	C_12_H_26_	17.81	33.78 ± 2.14c	10.64 ± 0.51d	—	105.36 ± 5.00b	228.00 ± 9.42a	112-40-3
70	1-Methyl-2-(1-methylpentyl)-cyclopropane	C_10_H_20_	17.95	16.81 ± 1.59a	14.27 ± 0.52b	—	—	—	1000222-86-6
71	1-Methyl-2-ethyl-3-propyl-cyclobutane	C_10_H_20_	18.67	—	16.17	—	—	—	61233-72-5
72	5-Methyl-3-undecene	C_12_H_24_	18.81	10.06 ± 0.60a	9.37 ± 0.58a	—	—	—	1000061-84-1
73	1-Methyl-2-propyl-cyclopentane	C_9_H_18_	18.81	—	15.53	—	—	—	3728-57-2
74	1-Methyl-4-(2-hydroxyethyl)-cyclohexane	C_9_H_18_O	18.98	—	—	109.97	—	—	4916-87-4
75	1-(2-Methylbutyl)-1-(1-methylpropyl)-cyclopropane	C_12_H_24_	19.71	—	—	17.18	—	—	64723-36-0
76	Tridecane	C_13_H_28_	20.22	13.08 ± 1.50b	9.61 ± 0.51b	—	103.46 ± 4.04a	113.92 ± 5.05a	629-50-5
77	3,6-Dimethyl-Undecane	C_13_H_28_	20.22	—	15.11	—	—	—	17301-28-9
78	(Z)-1,1,3,4-Tetramethyl-cyclopentane	C_9_H_18_	20.22	—	10.07	—	—	—	53907-60-1
79	4,8-Dimethyl-undecane	C_13_H_28_	20.22	—	—	20.15	—	—	17301-33-6
80	3,7-Dimethyl-undecane	C_13_H_28_	20.22	—	—	—	8.88	—	17301-29-0
81	5-Methyl-5-propyl-nonane	C_13_H_28_	20.22	—	—	25.71	—	—	17312-75-3
82	1-Isocyano-3-methyl-benzene	C_8_H_7_N	20.80	8.13 ± 0.61b	—	15.47 ± 1.59a	17.11 ± 1.54a	16.15 ± 0.94a	20600-54-8
83	2,6,10-Trimethyl-dodecane	C_15_H_32_	21.73	9.15 ± 0.94b	10.02 ± 1.01b	18.44 ± 1.46a	—	—	3891-98-3
84	Tetradecane	C_14_H_30_	22.20	31.99 ± 2.58bc	24.89 ± 1.78cd	49.92 ± 3.66a	33.31 ± 2.08b	18.76 ± 1.28d	629-59-4
85	2,6,10-Trimethyltridecane	C_16_H_34_	23.26	—	—	16.11 ± 1.54ab	11.36 ± 1.42b	16.51 ± 2.22a	3891-99-4
86	Pentadecane	C_15_H_32_	23.92	11.56 ± 0.58c	7.18 ± 0.65c	29.38 ± 3.09a	19.94 ± 1.22b	11.83 ± 0.61c	629-62-9
**Nitrogen- containing**									
87	Benzadehyde o-benzyloxime	C_14_H_13_NO	9.34	—	—	—	—	254.05	1000144-83-7
88	3-Methyl-1H-1,2,4-triazole	C_3_H_5_N_3_	9.59	7.63	—	—	—	—	7170/1/6
89	2,2'-azobis[2-Methyl-propanenitrile]	C_8_H_12_N_4_	9.94	—	405.31	—	—	—	78-67-1
90	Cyclohexyl(2-methylcyclohexyl)-propanedinitrile	C_16_H_24_N_2_	9.96	590.59	—	—	—	—	74764-55-9
91	2-Methyl-hexanedinitrile	C_7_H_10_N_2_	10.42	—	158.51	—	—	—	16525-39-6
92	Furfurylmethylamphetamine	C_15_H_19_NO	12.54	7.46	—	—	—	—	13445-60-8
93	Methoxy-phenyl-oxime	C_8_H_9_NO_2_	15.89	229.72 ± 3.65c	385.41 ± 21.94a	318.75 ± 9.98b	121.76 ± 8.50d	133.08 ± 8.52d	1000222-86-6
94	5-(Methylthio)-pentanenitrile	C_6_H_11_NS	18.57	52.67 ± 6.13d	58.83 ± 4.82d	91.18 ± 9.41c	346.57 ± 9.50a	150.07 ± 11.03b	59121-25-4
95	4-(Methylthio)-butanenitrile	C_5_H_9_NS	19.85	726.75 ± 4.00b	560.40 ± 25.91c	1472.88 ± 70.64a	—	—	59121-24-3
96	N,N-Dibutyl-formamide	C_9_H_19_NO	23.50	8.43 ± 0.66b	12.26 ± 1.58b	16.53 ± 1.24a	—	—	761-65-9
97	3-Phenylpropanenitrile	C_9_H_9_N	24.41	26.65 ± 2.01cd	18.83 ± 1.52d	45.11 ± 3.06c	68.11 ± 10.02b	89.73 ± 8.68a	645-59-0
98	5-Methyl-indolizine	C_9_H_9_N	25.72	—	—	—	13.43	—	1761-19-9
**Others**									
99	Acetic anhydride	C_4_H_6_O_3_	1.72	—	—	87.47	—	—	108-24-7
100	Dimethyl ether	C_2_H_6_O	2.07	—	135.88 ± 7.44b	321.50 ± 15.09a	97.27 ± 6.41c	151.72 ± 11.70b	115-10-6
101	2,2',3',5-Tetrahydro-2,3'-bifuran	C_8_H_10_O_2_	7.28	—	—	36.93	—	—	98869-93-3
102	Propanoic acid, anhydride	C_6_H_10_O_3_	7.54	—	—	—	73.89	—	123-62-6
103	Non-anoic acid 2-phenylethylester	C_17_H_26_O_2_	9.70	54.68	—	—	—	—	57943-67-6
104	(Z)-3-Hexen-1-ol, formate	C_7_H_12_O_2_	10.41	—	239.56	—	—	—	33467-73-1
105	Dimethyl trisulfide	C_2_H_6_S_3_	12.43	21.22 ± 1.74c	28.44 ± 3.12c	36.36 ± 6.63bc	48.65 ± 7.31b	103.21 ± 4.97a	3658-80-8
106	2-Pentyl-furan	C_9_H_14_O	12.54	—	—	—	—	10.78	3777-69-3
107	2-Propyl-furan	C_7_H_10_O	15.99	40.51 ± 3.03b	—	55.18 ± 3.10a	—	—	4229-91-8
108	2-Methoxy-phenol	C_7_H_8_O_2_	18.58	—	—	—	15.89 ± 1.59a	15.78 ± 0.95a	90-05-1
109	1,4-Dimethoxy-benzene	C_8_H_10_O_2_	19.58	—	—	—	9.06	—	150-78-7
110	1,2-Dimethoxy-benzene	C_8_H_10_O_2_	19.63	—	—	—	16.20 ± 0.64a	13.78 ± 1.17a	91-16-7
111	Valeric anhydride	C_10_H_18_O_3_	20.41	—	5.67 ± 0.55b	21.33 ± 2.57a	8.93 ± 0.83b		2082-59-9
112	Tetrasulfide, dimethyl	C_2_H_6_S_4_	20.52	—	—	—	—	12.59	5756-24-1
113	2-Hexyl-furan	C_10_H_16_O	23.20	—	—	—	22.80 ± 3.52b	30.07 ± 2.06a	3777-70-6
114	4-(1-Methylpropyl)-phenol	C_10_H_14_O	23.28	24.77 ± 2.71b	22.81 ± 2.56b	46.21 ± 4.52a	44.12 ± 4.74a	37.01 ± 2.65a	99-71-8
115	2-(1-Methylpropyl)-phenol	C_10_H_14_O	23.28	—	—	—	33.51	—	89-72-5
Total content/(μg/kg)			4,209.15 ± 23.13d	4,458.53 ± 126.078cd	6,867.59 ± 366.38a	4,792.04 ± 302.14bc	5,169.63 ± 287.34b	

“*-”, Not detected*.

*Values are expressed as the mean ± SE (n = 3). Different letters denote significant differences (p < 0.05). CK1, CK2, T1, T2, and T3 are defined in Figure 1*.

#### Effects of Different Ground Mulching Patterns on the Amounts and Relative Concentration of Volatile Compounds in Loose-Curd Cauliflower Heads

**Figure 3:**
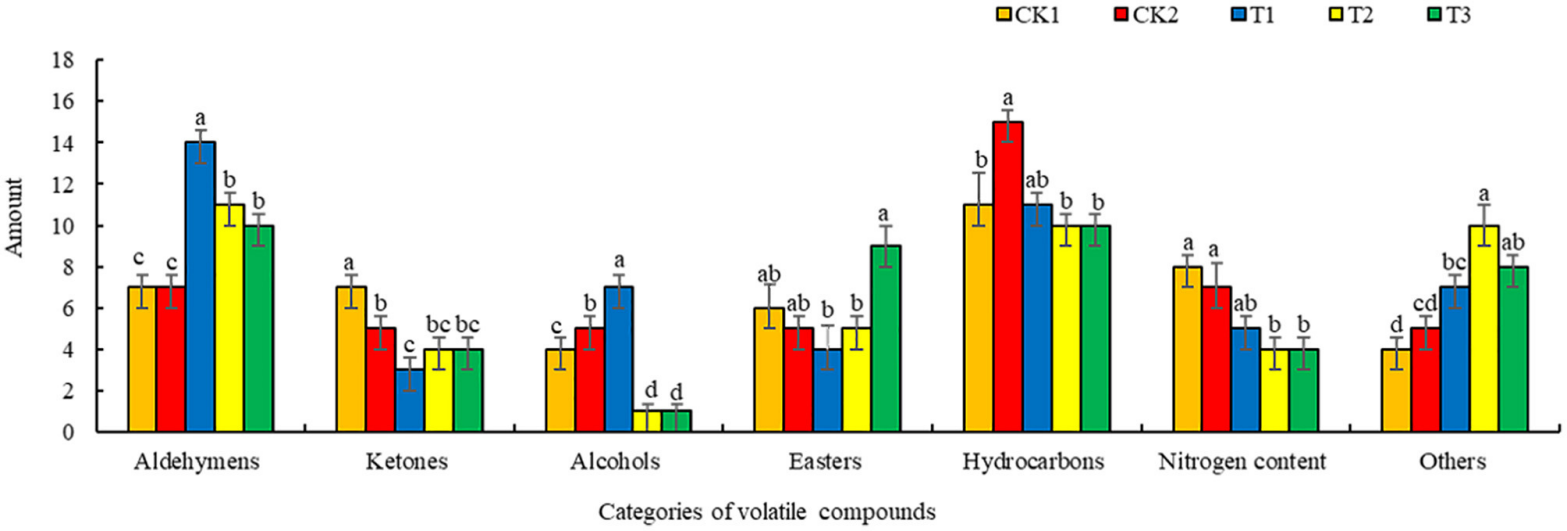
Effects of different ground mulching patterns on the amounts of volatile compounds in loose-curd cauliflower heads. Values are expressed as mean ± SE (*n* = 3). Different letters denote significant differences (*p* < 0.05). CK1, CK2, T1, T2, and T3 are defined in Figure 1.

Quantitatively, a total of seven chemical families, were detected in loose-curd cauliflower heads, as shown in Figure 3. These included mainly aldehydes (7–14 types), followed by 11–13 types of hydrocarbons, along with ketones, esters, nitrogen-containing and other compounds, and alcohols (of which the least types-−1-7—were detected.) Compared with CK1 and CK2 treatments, T1 treatment significantly increased the number of aldehydes and alcohols detected in the cauliflower. Cauliflower grown in T1-treated soil had the most aldehydes and alcohols, while those from T1-, T2, and T3-treated soil revealed lower numbers of ketones, hydrocarbons, and nitrogen-containing compounds, whereas the amount of esters in cauliflower grown under T3 treatment, was significantly increased. Additionally, the amount of esters in cauliflower grown under T1 and T2 treatments was lower, but there was no significant difference. The amounts of other compound types in cauliflower grown in T2-treated soil, were significantly higher than in those grown under the other treatment.

**Figure 4:**
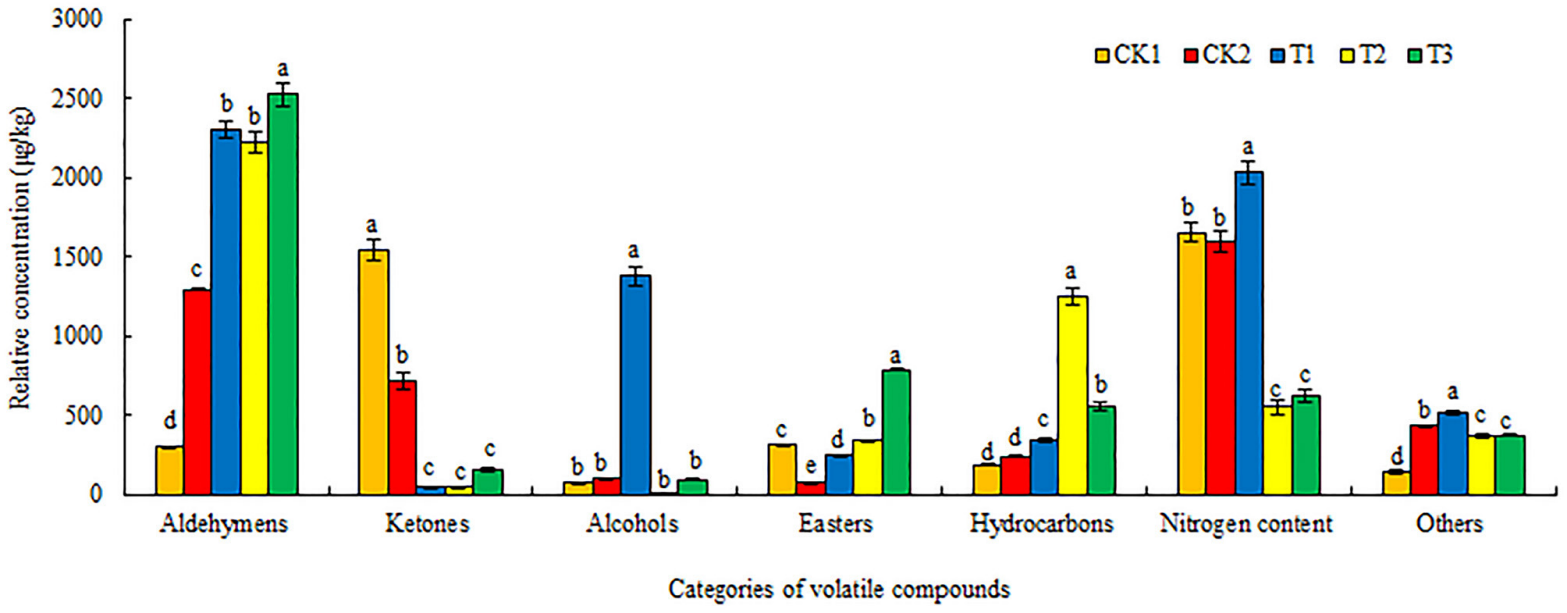
Effects of different ground mulching patterns on the relative concentration of volatile compounds in loose-curd cauliflower heads. Values are expressed as mean ± SE (*n* = 3). Different letters denote significant differences (*p* < 0.05). CK1, CK2, T1, T2, and T3 are defined in Figure 1.

As shown in Figure 4, significant differences were observed in the relative concentration of volatile compounds in loose-curd cauliflower heads, grown in soil subjected to the five different treatments. Aldehyde (297.31–2526.24 μg/kg) and nitrogen (549.87–2031.92 μg/kg) contents were higher, followed by ketones (45.42–1542.47 μg/kg). The contents of alcohols (10.48–1378.57 μg/kg) hydrocarbons (188.06–1244.64 μg/kg), and esters (73.44–788.39 μg/kg) were lowest. Compared with cauliflower grown under CK1 and CK2 treatments, those from soil subjected to T1, T2, and T3 treatments revealed significantly increased aldehyde content, of which those from T3-treated soil had the highest, showing increases of 749.71 and 95.2%, respectively. T1 treatment resulted in the lowest ketone content, whereas T2 and T3 treatment resulted in significantly higher esters and hydrocarbons than the CK1 and CK2 treatments. T3 treatment produced the highest ester content, T2 treatment resulted in the highest content of hydrocarbons, and T1 treatment brought about significantly more abundant nitrogen-containing substances, alcohols, and other compounds than the CK1 and CK2 treatments.

#### Effects of Different Ground Mulching Patterns on Common and Specific Volatile Compounds in Loose-Curd Cauliflower Heads

**Figure 5:**
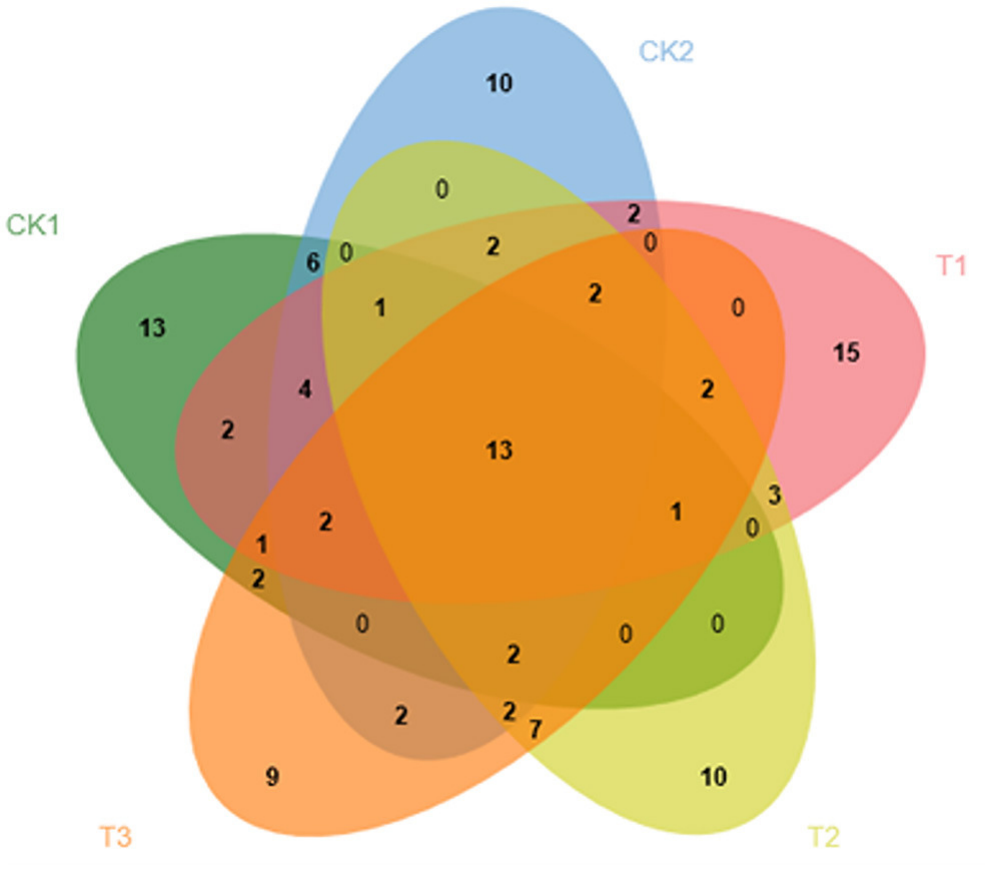
Effects of different ground mulching patterns on common and specific volatile compounds in loose-curd cauliflower heads.

As shown in Figure 5, only 13 types of volatile substances were common among the 5 treatments, indicating that different ground cover methods have a major impact on the types of volatile compounds detected in loose-curd cauliflower heads. The 13 common compounds comprised 5 aldehydes, 1 ester, 2 hydrocarbons, 3 nitrogen-containing substances, and 2 other substances; no ketones or alcohols were detected. Methoxy-phenyl-oxime was most abundant among the common substances, reaching 385.41 μg/kg. CK1, CK2, T1, T2, and T3 treatments produced 13, 10, 15, 10, and 9 specific compounds, respectively. Among the 13 substances unique to the CK1 treatment, there were 4 ketones, 1 alcohol, 3 esters, 1 hydrocarbon, 3 nitrogen-containing substances, and 1 other substance, but no aldehydes. Of these, cyclohexyl(2-methylcyclohexyl)-propanedinitrile content was the highest, reaching 590.59 μg/kg. The 10 substances unique to CK2 treatment, included 1 ketone, 1 alcohol, 5 hydrocarbons, 2 nitrogen-containing substances, and 1 other substance, but 0 aldehydes or esters. At a maximum of 405.31 μg/kg, 2,2'-azobis[2-methyl-propanenitrile] was the most abundant substance specific to CK2 treatment. T1 treatment produced 3 aldehydes, 1 ketone, 4 alcohols, 1 ester, 5 hydrocarbons, and 2 other substances, but 0 nitrogen-containing compounds, as part of the 15 substances unique to T1 treatment. (E)-4-hexen-1-ol was the substance with the highest content, of those substances specific to T1 treatment, reaching 472.91 μg/kg. T2 treatment brought about 1 type of aldehyde, 2 ketones, 1 nitrogen-containing compound, 3 hydrocarbons, and 3 other substances among the 10 types of substances unique to T2 treatment; 0 alcohols and esters were detected. 4-methyl-3-pentenal was the substance with the highest content, reaching 1112.77 μg/kg. T3 treatment produced 9 unique substances, including 1 aldehyde, 1 ketone, 2 esters, 1 nitrogen-containing compound, 2 hydrocarbons, and 2 other substances, but 0 alcohols. Of these ethyl (E)-hex-3-enyl carbonate was most abundant, reaching 440.46 μg/kg.

#### Principal Component Analysis of Different Coverage Treatments

**Figure 6:**
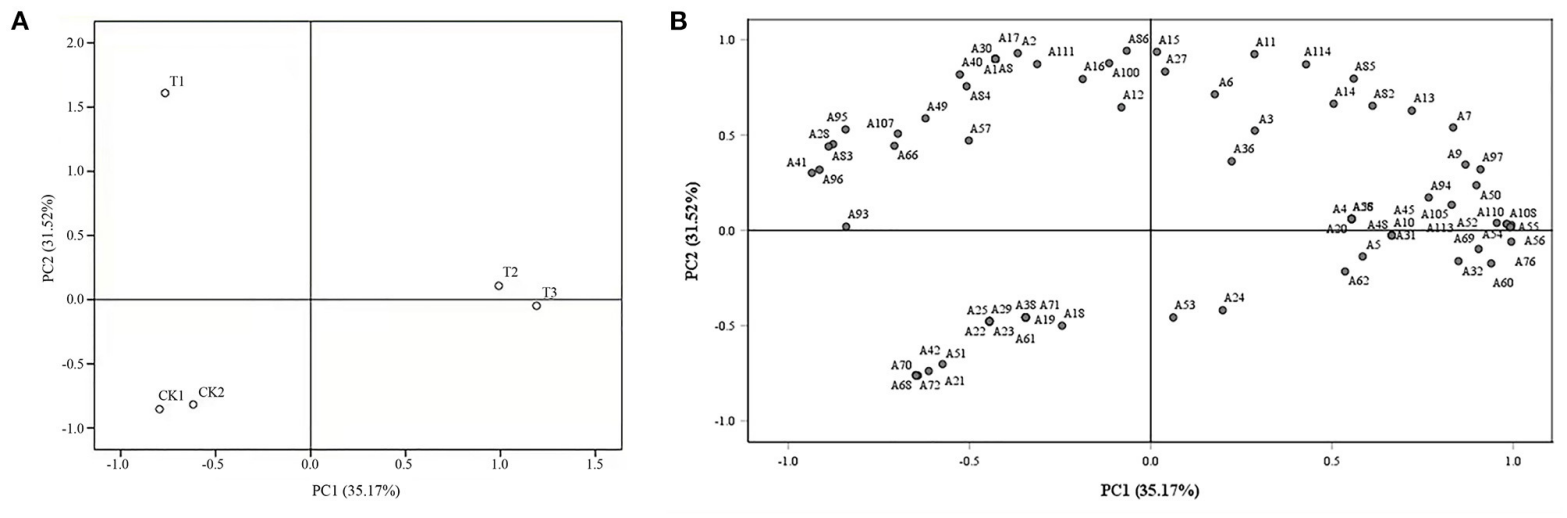
Principal component analysis (PCA) of loose-curd cauliflower heads and 115 volatile compounds. **(A)** shows the PCA loading plot, whereas **(B)** shows the PCA scatter plot. The number code after “A” in **(B)** also corresponds to the relevant volatile code in Table 4.

The PCA loading plot of the different treatments is shown in Figure 6A. The sum of the first two principal components reached 66.69%, of which PC1 and PC2 represented 35.17 and 31.52% of the total variance, respectively. Treatments were divided into three groups. The second principal component sorted the T1 treatment into a group and was located in the second quadrant, whereas the CK1 and CK2 treatments were the first and second principal components, close to one group and located in the third quadrant. For the T2 and T3 treatments, the first and second principal components were close to one group and located in the first and fourth quadrants, respectively.

Figure 6B reveals that (E)-2-hexenal (A3), 4-(methylthio)-butanenitrile (A95), and 4-methyl-3-pentenal (A4) were the three volatile substances with the highest content in loose-curd cauliflower heads, at 1565.9, 1472.88, and 1112.77 μg/kg, respectively. The T1 treatment, which formed a separate group, was mainly enriched in 1,5-pentanedial (A1), 1-hepten-3-one (A2), (E)-4-oxohex-2-enal (A8), 1-decanal (A15), (Z)-4,5-epoxy-2-decenal (A17), 1-hepten-3-one (A30), (S)-(+)-5-Methyl-1-heptanol (A40), pentadecane (A86), dimethyl ether (A100), and valeric anhydride (A111). Both the T2 and T3 treatments contained high amounts of benzaldehyde (A7), (E,E)-2,4-heptadienal (A9), 1-nonanal (A13), octyl formate (A50), 3-methylthiopropyl isothiocyanate (A54), erucin (A55), (2-isothiocyanatoethyl)-benzene (A56), 2-methoxy-phenol (A108), and 1,2-dimethoxy-benzene (A110). Moreover, both CK1 and CK2 treatments resulted in high levels of 3-methyl-cyclopentanone (A21), (S)-(+)-6-methyl-1-octanol (A42), and (Z)-4-methyl-2-decene (A68).

## Discussion

Because of the long-term excessive application of chemical fertilizers, deficient soil organic matter, soil compaction, and reduced soil water, fertilizer utilization and in output have severely restricted the development of agriculture in arid and semi-arid areas (36–38). However, straw mulching can increase the organic matter content in soil and improve both soil water use efficiency and crop yield (39, 40). Dry matter represents the structures by which crops absorb nutrients and conduct photosynthesis, of which the accumulated products are reasonably distributed, which is conducive to increasing crop production. Studies have shown that straw mulching can increase corn kernel yield by 16.40%, by increasing the contribution rate of the various organs of the corn plant (41). This study found that dual mulching with straw and mulching film (T1 treatment) promoted the accumulation of dry matter during the entire growth period, as measured at different points in the growth cycle, compared with plastic film mulch only (CK2). The dry weight of the above- and below ground plant structures, increased by 21.01 and 18.12%, 10.24 and 17.37%, and 11.83 and 16.57%, respectively (Table 2). Dual mulching with straw and mulching film (T1) promoted the accumulation of dry matter, which translated into increased yield. A previous study in Northwest China has indicated that a combination of straw mulching with no-tillage and plastic film mulching substantially increased corn yield, by 13.00% (42). Our study also found that dual mulching with straw and mulching film (T1) could increase the economic and biological yield of loose-curd cauliflower by 12.98 and 6.51%, respectively, compared with plastic film coverage only (CK2). Moreover, the yield of loose-curd cauliflower grown in soil treated with inter-row straw mulch (T2) and full straw mulch (T3), was significantly reduced (Table 3). On the one hand, this may be due to the poor heat preservation and water retention effect of inter-row straw mulch (T2). On the other hand, full straw mulching (T3) lowers the growth temperature of crops at the seedling stage, delaying crop growth, which is not conducive to the accumulation of dry matter and reduces output. Another previous study has found that straw mulching throughout the year could increase the yield of dryland wheat in China, and achieve increased yield and efficiency (12).

Excessive application of fertilizers causes vegetable quality to decline. Studies have shown that drip irrigation, combined with straw mulching, can substantially increase the sugar, vitamin C, and lycopene content in tomato fruit (43), similar to the results of the present study. It has further been shown that straw mulching can substantially increase the protein content of rice, reduce the content of brown rice, effectively balance rice quality indicators, and notably improve the overall quality of rice (44). Our results showed that dual mulching with straw and plastic film (T1) significantly improved the quality of loose-curd cauliflower heads, compared with plastic film mulching only (CK2). Accordingly, the soluble sugar and vitamin C content increased significantly—by 18.46 and 8.12%, respectively—while the nitrate content was significantly reduced by 25.57% (Figure 2). The reason may be that the return of straw to the field increases the organic matter content in the soil, which improves the quality of vegetables.

The accumulation of mineral elements is of great significance for improving the quality of vegetables. Our study showed that the macro- (P, K), meso- (Ca, Mg), and micro- (trace) elements (Fe, Mn, Zn) in the loose-curd cauliflower heads grown under T1, T2, and T3 treatments had increased, of which those from soil subjected to full straw mulch (T3) treatment, revealed the greatest increase (Table 4). This is ascribed to straw mulch improving soil fertility and promoting the accumulation of mineral elements. Researchers have shown that straw mulching treatment increases the N, P, and K content of spinach, throughout its growing season. This may be because straw is rich in these elements, and straw mulching promotes their absorption by spinach (40). It has further been indicated that, compared with clear tillage, straw mulching treatment substantially increases macro-, meso-, and microelement content in apples (45), correlating with the results of this study. Other studies have indicated that straw mulching treatment increases the mineral element content in potato tubers, compared with no mulching (46).

The results of this experiment showed that straw mulching treatment significantly increased the volatile compound content in loose-curd cauliflower heads, while the volatile compound content and quantity increased most significantly in soil subjected to dual mulching with straw and plastic film (T1) (Figures 3, 4). This may be because the return of straw to the field increases the organic matter content in soil, which, in turn, could increase the types and content of volatile compounds (47). Our results showed that 115 volatile compounds were detected among the 5 treatments prepared in this experiment, which mainly comprised aldehydes, ketones, alcohols, esters, hydrocarbons, and other compounds (Table 5). Glucosinolates—types of sulfur-containing secondary metabolites—are unique to cruciferous vegetables. When plant cells are damaged, myrosinase is released and converts glutamine into various volatile compounds, including isothiocyanates and nitriles, which have antibacterial and anti-inflammatory functions, as well as anticancer effects. Additionally, isothiocyanates provide vegetables with a pungent odor, which is very important for food sensory characteristics (48–50). This experimental study showed that 1-isothiocyanato-3-(methylthio)-propane, erucin, (2-isothiocyanatoethyl)-benzene, and 5-(methylthio)-pentanenitrile, the contents of 4-(methylthio)-butanenitrile and 3-phenylpropanenitrile, had been increased to varying degrees (Table 5), indicating that straw mulching treatment aided the decomposition of glucosinolates in loose-curd cauliflower heads. Moreover, straw mulching might maintain a suitable temperature of the cultivated layer, improve the activity of myrosinase, and promote the decomposition of glucosinolates into nitriles and isothiocyanates (51). Among the treatment preparations in this experiment, dual mulching with straw and mulching film (T1) elicited the most types of volatile substances in loose-curd cauliflower heads. Further, major differences were detected in the absolute content of volatile compounds between cauliflower grown under straw mulching treatment and those grown in an open field without mulching (CK1) or with plastic film mulching (CK2). The content of aldehydes and esters in cauliflower grown under the straw mulching treatment was higher, whereas dual mulching with straw and plastic film (T1) significantly increased the content of alcohols, in particular; T1 treatment uniquely elevated the content of four alcohols, which were (S)-(+)-1,2-propanediol, (E)-4-hexen-1-ol, (E)-3-hexen-1-ol, and 2,3-butanediol, amounting to 365.49 μg/kg, 472.91, 138.59, and 276.00 μg/kg respectively (Figure 4).

β-ionone, 2-phenylethanol, methyl salicylate, and 2-isobutylthiazole were not detected in the loose-curd cauliflower heads analyzed in this study, which may have been caused by factors such as variety analyzed, cultivation conditions, and the environment (25, 52). However, dimethyl trisulfide—a volatile compound in loose-curd cauliflower that produces a pungent odor—as well as dimethyl tertrasulfide, with the smell of garlic, were detected. These two compounds are also detected in cabbage and other cauliflower varieties, and their content is typically higher in vegetables of the *Brassica* species (35, 53). The results of this experiment revealed 13 and 10 unique compounds, respectively, in cauliflower grown in the open field without mulch (CK1) and those grown under plastic film mulch (CK2) treatment, compared with 15 unique compounds under the straw and plastic dual mulch (T1) treatment. Moreover, 10 unique compounds were detected under inter-row straw mulch (T2) and 9, under full straw mulch (T3), indicating that the dual mulch (T1) technique had the greatest impact on accumulation of volatile components in loose-curd cauliflower heads. The results showed that 13 types of volatile compounds—mainly comprising aldehydes and hydrocarbons—were detected among the different ground cover treatments, indicating that the ground cover method had a greater impact on the volatile compounds in loose-curd cauliflower heads (Figure 5), than the differences in water, temperature, and soil fertility between different treatments, which were speculated to have a certain effect. The aromatic compounds in loose-curd cauliflower heads detected in this study, were mainly green, fruity, and floral. Green aroma compounds were the most abundant, of which (E)-2-hexenal had the highest content, presenting a fruity aroma. Others have also detected this compound, which is an effective flavor component, in cabbage heads (35).

The potential volatile compounds in loose-curd cauliflower heads were analyzed using the scatter diagram and loading graph, to find different compounds and comprehensively evaluate the influence of different ground covering methods on these volatile compounds. Subsequent to PCA, data could be divided into three groups; the first and second principal components of CK1 and CK2 were similar and sorted into one group, whereas, in the T2 and T3 treatments, the first and second principal components were close to one group, and the second principal component sorted the T1 treatment into a separate group (Figure 6A). The scatter plot—through which the characteristic volatile substances in loose-curd cauliflower heads were further explored—indicated that (E)-2-hexenal (A3), 4-(methylthio)-butanenitrile (A95), and 4-methyl-3-pentenal (A4) were characteristic volatile substances of loose-curd cauliflower heads. T1 treatment enriched 1,5-pentanedial (A1), 1-hexanal (A2), (E)-4-oxohex-2-enal (A8), 1-decanal (A15), (Z)-4,5-epoxy-2-decenal (A17), 1-hepten-3-one (A30), (S)-(+)-5-methyl-1-heptanol (A40), pentadecane (A86), dimethyl ether (A100), and valeric anhydride (A111) (Figure 6B). Few reports on the flavor compounds in loose-curd cauliflower heads, exist and many related uncertainties remain to be solved. For example, the contribution of volatile compounds detected in loose-curd cauliflower heads to its flavor, is still unclear and the effect of straw mulching on improving the flavor of loose-curd cauliflower, needs to be explored. Evaluation of this mechanism would further provide a basis for the synthesis and adjustment of volatile compounds, and establish the relationship between the quality, yield, and volatile compounds in loose-curd cauliflower. In turn, this would provide a theoretical basis and technical support for the production of high-yield and -quality, open field loose-curd cauliflower.

## Conclusion

The results of this study showed that dual mulching with straw and plastic film promoted the accumulation of dry matter in, increased the yield of, and improved the soluble sugar, protein, and vitamin C and mineral element content in loose-curd cauliflower, while significantly reducing the nitrate content. A total of 115 volatile compounds were identified in loose-curd cauliflower heads, using HS-SPME-GC-MS metho-dology, mainly aldehydes, ketones, alcohols, esters, hydrocarbons, nitriles and ethers. Dual mulching with straw and plastic film increased the total number and total content of volatile compounds in loose-curd cauliflower. Moreover, the number and content of alcohol and aldehyde substances increased significantly. In summary, the dual mulching with straw and plastic film could significantly improve the yield and quality of loose-curd cauliflower, and effectively improve the flavor of loose-curd cauliflower heads. This mulching technique could be applied in corn production areas to realize and theoretically support the production of high-quality, high-yield open field vegetables, as well as crop stalk recycling.

## Data Availability Statement

The original contributions presented in the study are included in the article/supplementary material, further inquiries can be directed to the corresponding authors.

## Author Contributions

JLy, JY, YX, and JLi conceived and designed the research. YX and JLi conducted the experiments. YX, JW, and NJ analyzed the data and prepared the figures and illustrations. YX wrote the manuscript. JLy, JY, LJ, SWe, SWa, JX, ZF, and GZ read the manuscript and made valuable inputs. All authors read and approved the submission of the manuscript.

## Funding

This research was funded by the Education Science and Technology Innovation Project of Gansu Province (GSSYLXM-02), National Modern Agricultural Industrial System Special Project (CAR-23-C-07), the Special project of Central Government Guiding Local Science and Technology Development (ZCYD-2021-07), Gansu People's Livelihood Science and Technology Project (20CX9NA099), Gansu Province Top Leading Talent Program (GSBJLJ-2021-14), and Gansu Provincial Department of Education: Excellent Postgraduate Innovation Star Project (2021CXZX-373).

## Conflict of Interest

The authors declare that the research was conducted in the absence of any commercial or financial relationships that could be construed as a potential conflict of interest.

## Publisher's Note

All claims expressed in this article are solely those of the authors and do not necessarily represent those of their affiliated organizations, or those of the publisher, the editors and the reviewers. Any product that may be evaluated in this article, or claim that may be made by its manufacturer, is not guaranteed or endorsed by the publisher.
